# Using curcumin to prevent structural impairments of testicles in rats induced by sodium metabisulfite

**DOI:** 10.17179/excli2017-143

**Published:** 2017-04-24

**Authors:** Reza Mahmoudi, Zahra Honarmand, Saied Karbalay-Doust, Mehrzad Jafari-Barmak, Mohsen Nikseresht, Ali Noorafshan

**Affiliations:** 1 Cellular and Molecular Research Center, Yasuj University of Medical Sciences, Yasuj, Iran; 2 Student Research Committee, Yasuj University of Medical Sciences, Yasuj, Iran; 3 Anatomy Department, School of Medicine, Shiraz University of Medical Sciences, Shiraz, Iran; 4 Histomorphometry and Stereology Research Center, Shiraz University of Medical Sciences, Shiraz, Iran

**Keywords:** curcumin, rat, sodium metabisulfite, stereology, testicle

## Abstract

Sodium metabisulfite (Na-MBS) is a disinfectant and preservative agent. Some organ including testicle would be in danger in the case of Na-MBS consumption. Curcumin (CUR) is the constituent of turmeric with protective properties. The effect of CUR on testicles in rats exposed to Na-MBS evaluated using stereological methods. Sprague-Dawley rats were divided into eight groups. The rats in groups I to VIII received the following respectively: distilled water, CUR (100 mg/kg/day), low (0.7 mg/kg/day: acceptable daily intake), intermediate (7 mg/ kg/day), and high (70 mg/kg/day) doses of Na-MBS, and low, intermediate, and high doses of Na-MBS plus CUR. After 7 weeks, the testicles were analyzed. The volume of seminiferous tubule, tubular epithelium and tubule length reduced (25-40 %) on average in the rats that received intermediate and high doses of Na-MBS, while the connective tissue volume increased (15-20 %) in both groups (P<0.01). Besides, 19-36 % and 41-57 % of the cells (spermatogonia types A and B, spermatids, Sertoli and Leydig) were lost in the rats that received intermediate and high doses of Na-MBS respectively in comparison to the control groups. Nonetheless, all the above-mentioned alterations ameliorated drastically in the rats that received Na-MBS plus CUR compared to those exposed to Na-MBS without CUR therapy (P<0.01). The acceptable daily intake of Na-MBS for 7 weeks did not affect on testicular parameters. CUR (100 mg/kg/day) could prevent structural impairments of testicles in the rats induced by Na-MBS (7 and 70 mg/kg/day).

## Introduction

Sulfite salts including sodium metabisulfite (Na-MBS) are often used as disinfectants, antioxidants as well as preservative compounds in food such as pastries, cheese, beverages, meat, fruit, sausages, sweets, and fish (Elmas et al., 2005[9]). This serves to decelerate the growth of bacteria, mould, and yeasts (Ercan et al., 2015[10]). Once ingested, these salts react with water, leading to formation of bisulfite, sulfite, and sulfur dioxide. An enzyme named sulfite oxidase is responsible for detoxification of SO_2_. Different tissues exhibit different sulfite oxidase activities. For instance, liver and kidney show high, whereas testicle shows very low sulfite oxidase activities (Cabré et al., 1990[7]; Woo et al., 2003[23]). The majority of previous work has only focused on the effects of sulfating agents of these structures on the brain, while few studies have been published on these effects on the testicle. In addition, these limited researches have not evaluated the testicle using quantitative microscopic stereological methods.

The Acceptable Daily Intake (ADI) for Na-MBS is considered to be 0.7 mg/kg body weight (Nair and Elmore, 2003[15]). However, the exact intake varies depending on individuals' dietary habits. Given that individuals' exact intakes are not clear throughout the day in diverse dietary habits, two other doses, including intermediate and high, were defined as 10 and 100 times of ADI. Thus, three doses of Na-MBS were introduced in the present study. The low, intermediate, and high doses of Na-MBS were considered to be 0.7, 7, and 70 mg/kg/day respectively. 

The main aim of this study is to evaluate the effects of different doses of Na-MBS on the quantitative structural aspects of the testicle. Secondly, it aims to introduce a protective agent that could be easily consumed with the lowest adverse effects. It has been reported that turmeric has some beneficial effects on reproductive disorders (Joe et al., 2004[12]). To prevent the confusing interpretation of the results by several interactive components of turmeric, its main constituent named Curcumin (CUR) was considered as the protective agent in this study. In the literature there are some examples of antiviral, anti-infective, and antioxidant properties of CUR (Araújo and Leon, 2001[5]). It has also been shown that CUR is able to prevent oxidative changes in sperms and testicular tissues, thereby improving sperm motility and diminishing spermatozoa abnormalities (Farombi et al., 2007[11]). The selected dose of CUR in the present study, 100 mg/kg/day, is based on previous studies, which showed the protective effects of the agent on the testicular tissue (Takhtfooladi et al., 2015[20]; Sharma and Singh, 2010[18]).

In an attempt to find the answers to the following hypothetical queries, the present survey was conducted on a rat model consuming ADI, intermediate and high doses of Na-MBS:

Does sperm quality change after the ingestion of Na-MBS? Does the volume of testicle (connective tissues and seminiferous tubules) change after the treatment with Na-MBS? Does the number of spermatogenic cells (spermatogonia, spermatocytes and spermatids cells) change after consuming Na-MBS? Does the number of Sertoli and Leydig cells change after ingesting Na-MBS? Does the length of tubules alter after exposure to Na-MBS? Can CUR prevent the alteration in sperm quality caused by the treatment with Na-MBS? Can CUR prevent changes in the testicular structure in animals? 

To detect structural changes in the testicle, the tissue was assessed using stereological methods. Unbiased stereological methods could help obtain quantitative, reliable, and comparable data. 

## Materials and Methods

### Animals

In this study, we opted for a rat model consisting of 56 male Sprague-Dawley rats weighing 210-270 g which were obtained from the Center of Comparative and Experimental Medicine of the University. All animal procedures were performed under the standard rules established by the Animal Care and Ethics Committee of the University (agreement license No. 23-2-555).

### Experimental design

The rats were first randomly divided into eight experimental groups each containing 7 animals. Through daily gavage feeding, the animals in the groups received the following for a period of 7 weeks:

- Group I: Control (distilled water)
- Group II: CUR (100 mg/kg/day)
- Group III: Low dose of Na-MBS(0.7 mg/kg/day)
- Group IV: Intermediate dose of Na-MBS (7 mg/kg/day)
- Group V: High dose of Na-MBS (70 mg/kg/day)
- Group VI: Low dose of Na-MBS (0.7 mg/kg/day) and CUR (100 mg/kg/day)
- Group VII: Intermediate dose of Na-MBS (7 mg/kg/day) and CUR (100 mg/kg/day)
- Group VIII: High dose of Na-MBS (70 mg/kg/day) and CUR (100 mg/kg/day)

Distilled water and phosphate buffer were the solvents of Na-MBS and CUR respectively. The low dose of Na-MBS was selected according to the ADI, which is 0.7 mg/kg/day (Elmas et al., 2005[9]; Sharma and Singh, 2010[18]; Rashid and Sil, 2015[16]).

### Spermatozoa counts, morphology and motility

The rats' ductus deferens were evaluated according to our previous research (Aminsharifi et al., 2016[4]).

### Stereological study 

**Figure 1:**
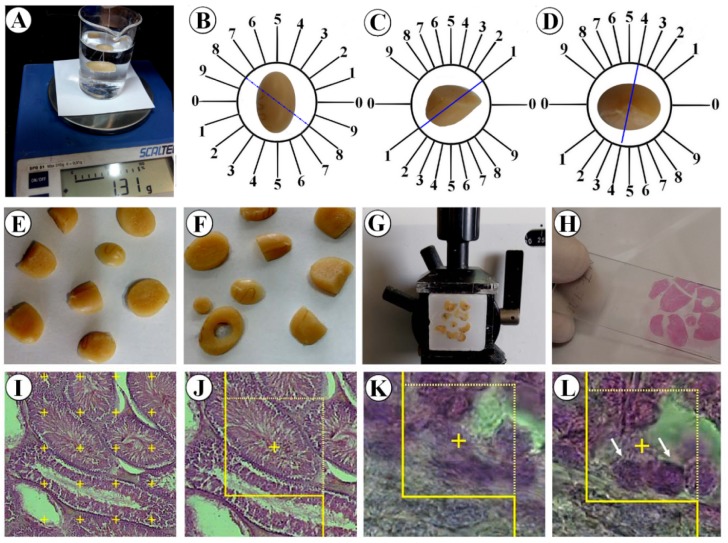
Application of stereological techniques. A. Immersion method. B. Obtaining isotropic uniform random sections by slicing the testicle according to the random direction of the evenly divided circle. C and D. Slicing each half of the testicle according to the random direction of the cosine-weighted divided circle. E. Obtaining a collection of isotropic uniform random sections. F. Punching out a circle through a random slice. G. Embedding and sectioning. H. Tissue slide preparation. I. Point-counting technique to estimate the volume density of the structures. J. Estimation of the length density of the seminiferous tubules by unbiased counting frame. K and L. Two optical sections of the testicular tissue to obtain the numerical density of different cells.

On the last day of the trial, the testicle was dissected out and weighed. Then, its primary volume “V (testicle)” was measured using the immersion method according to the method of Scherle (1970[17]) (Figure 1(Fig. 1)). The time-consuming consecutive sectioning of the testicle, which is needed for estimation based on Cavalieri principle, was eluded by estimation of the degree of shrinkage “d (shr)”. Estimating the “d (shr)” and tubule length requires isotropic uniform random sections. These sections were prepared according to the “orientator method” (Figure 1(Fig. 1)) (Dorph-Petersen et al., 2001[8]; Tschanz et al., 2014[21]; von Bartheld, 2012[22]; Arslan et al., 2016[6]). Totally, 8-12 slabs were collected from each testicle. Afterwards, a circle was punched out from a random testicle slab by a trocar and the area of the piece was calculated. Then, the slabs and circular pieces were processed, sectioned (4 and 25 µm thick), and stained with Heidenhain's Azan and Hematoxylin-Eosin (Figure 1(Fig. 1)) (Dorph-Petersen et al., 2001[8]; Tschanz et al., 2014[21]; von Bartheld, 2012[22]; Arslan et al., 2016[6]). The area of the circular piece and the degree of shrinkage “d (shr)” were calculated again:

*d (shr)= 1- (AA/AB)^1.5^*

where AA and AB represent the area of the circular piece after and before processing and staining.

The sections were analyzed using a video microscopy system. In doing so, the microscopic fields were sampled using a systematic random sampling procedure. Then, the stereological test grids (point grid and unbiased counting frame) were superimposed on the microscopic images on a monitor by means of the stereology software designed at the University (Figure 1(Fig. 1)).

### Estimation of the volume of the testicle components

The volume density “Vv(structure/testicle)” of the testicle (including tubules, germinal epithelium, and interstitial tissue) was estimated using “point-counting method” (Dorph-Petersen et al., 2001[8]; Tschanz et al., 2014[21]; von Bartheld, 2012[22]; Arslan et al., 2016[6]). The total volume of each structure was obtained using the following formula: 

V(structure) = Vv(structure/testicle) × V(testicle)

### Estimation of the cells number

The numerical density “Nv(cells/testicle)” and the total number of spermatogonia (types A and B), spermatocytes, spermatids (round and long), and Sertoli and Leydig cells were calculated using the “optical disector” method applied on the 25 µm thick sections. By means of the stereology software, a microcenter (Heidenhain MT-12, Leipzig, Germany) and a high numerical aperture oil immersion lens the “Nv(cells/testicle)” was estimated according to the “disector method” (Figure 1(Fig. 1)). After recording the distribution of all the sampled cells in different focal planes a plot was drawn for z-axis distribution to determine the guard zones and disector's height of the tissue section. The numerical density or the number of cells in the unit volume of germinal epithelium “Nv(cells/testicle)” was estimated using the following formula: 

*Nv(cells/testicle)= ΣQ/ (ΣA×h) × (t/BA)*

where ΣQ is the number of nuclei coming into focus, ΣA indicates the total area of the unbiased counting frame in all fields, “h” represents the “disector's height”, “t” is the mean section thickness, and finally BA indicates the microtome setting (Dorph-Petersen et al., 2001[8]; Tschanz et al., 2014[21]; von Bartheld, 2012[22]; Arslan et al., 2016[6]). The total number of the cells was estimated using this formula:

N(cells) = Nv(cells/testicle) ×V(epithelium) × [1−d(shr)]

### Estimation of tubules length 

The length density (Lv) of the tubules was calculated as (Figure1(Fig. 1)): 

*Lv= 2ΣQ / [ΣP× (a/f)]*

where “Σ*Q*” is the total number of the tubule profiles counted per rat testicle, “ΣP” represents the total number of frames counted in each animal, and “*a/f”* indicates the area of the counting frame. The total length of the tubules “L(tubules)” was calculated using the formula below (Dorph-Petersen et al., 2001[8]; Tschanz et al., 2014[21]; von Bartheld, 2012[22]; Arslan et al., 2016[6]):

*L(tubules)= L_V _(tubules/testicle) × [1−d(shr)]^ 2/3^ × V(testicle)*

### Statistical analysis

Statistical comparisons were made using Kruskal-Wallis and Mann-Whitney U tests. Furthermore, P<0.05 was considered to be statistically significant.

## Results

### Spermatozoa count, morphology and motility 

According to Table 1(Tab. 1), no change was identified in the parameters of spermatozoa in low dose Na-MBS treated (0.7 mg/kg/day) rats. However a significant respective decrease was observed in the number of spermatozoa, percentage of normal morphology spermatozoa and percentage of motile spermatozoa in the animals exposed to intermediate and high (7 and 70 mg/kg/day) doses of Na-MBS compared to the control groups (P<0.05). The count and normal morphology of the spermatozoa in the rats treated with intermediate and high of Na-MBS plus CUR improved in comparison with the related Na-MBS groups. No improvement was seen in motility of the spermatozoa after CUR co-treatment with Na-MBS. 

### Volume of the testicle 

**Figure 2:**
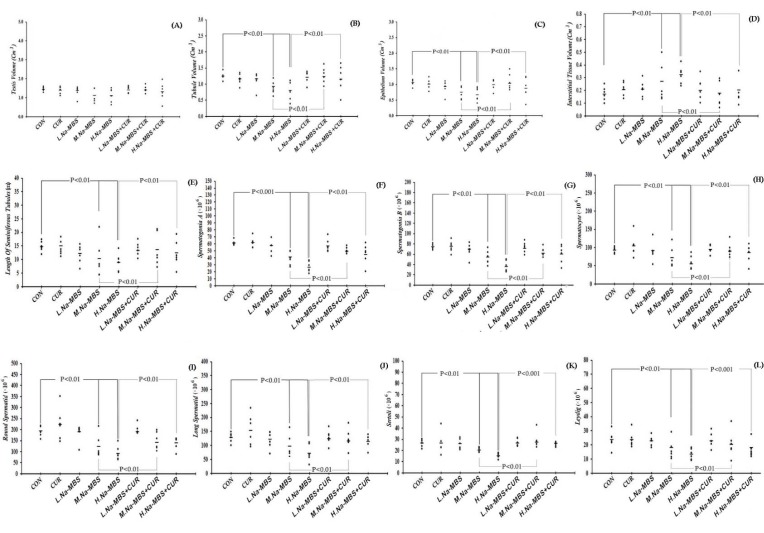
The scatter plots of the volumes of the testicle (A), tubules (B), epithelium (C), and interstitium (D), the length of seminiferous tubules (E), and the number of spermatogonia A (F), spermatogonia B (G), spermatocytes (H), round spermatids (I), long spermatids (J), Sertoli (K), and Leydig (L) in the control (CON), curcumin (CUR), low dose of Na-MBS (L. Na-MBS), intermediate dose of Na-MBS (M. Na-MBS), high dose of Na-MBS (H. Na-MBS), low dose of Na-MBS+CUR (L. Na-MBS+CUR), intermediate dose of Na-MBS+CUR (M. Na-MBS+CUR), and high dose of Na-MBS+CUR (H. Na-MBS+CUR) rats. Each dot represents an animal and the horizontal bar is the mean number of animals in each group. The significant differences are identified on each plot.

The results also revealed a negligible change in the testicle volumes in the groups that received intermediate and high doses of Na-MBS and Na-MBS plus CUR (Figure 2(Fig. 2)). 

### Volume of the seminiferous tubules

The tubules volumes reduced by 25 % and 26 % in the rats exposed to intermediate and high doses of Na-MBS respectively compared to the control groups (P<0.01). However, this parameter recovered considerably in the animals that received Na-MBS plus CUR compared to the Na-MBS groups (Figure 2(Fig. 2)). 

### Volume of the seminiferous tubules epithelium

The results also indicated that the total volume of the germinal epithelial decreased by respective values of 28 % and 36 % in the rats that received intermediate and high doses of Na-MBS as compared to the control rats (P<0.01). Yet, this reduction was recovered greatly in the Na-MBS plus CUR rats in comparison to the Na-MBS groups (Figure 2(Fig. 2)). 

### Volume of the connective tissue

It is also interesting to note that the connective tissue volume increased by 15 % and 20 % in the groups treated with intermediate and high doses of Na-MBS respectively in comparison to the control groups (P<0.01). Nevertheless, this significantly ameliorated in the rats that received Na-MBS (intermediate and high doses) plus CUR as opposed to those exposed to Na-MBS without CUR therapy (P<0.01) (Figure 2(Fig. 2)). 

### Length of the seminiferous tubules

The results also showed respective reductions of 30 % and 40 % in the tubules length of the rats that received intermediate and high doses of Na-MBS as compared to the control groups (P<0.01). Nonetheless, the tubules length greatly improved in the rats treated with Na-MBS (intermediate and high doses) plus CUR in comparison to those that received Na-MBS without CUR therapy (P<0.01) (Figure 2(Fig. 2)). 

### Number of cells

Analysis of the total number of testicular cells revealed significant losses in spermatogonia type A (33 %), spermatogonia type B (23 %), spermatocytes (19 %), round spermatids (36 %), long spermatids (24 %), Sertoli cells (24 %), and Leydig cells (24 %) in the animals exposed to the intermediate dose of Na-MBS as opposed to the control groups (Figure 2(Fig. 2)).

Further analysis of the total number of cells also indicated substantial losses in spermatogonia type A (57 %), spermatogonia type B (51 %), spermatocytes (41 %), round spermatids (53 %), long spermatids (44 %), Sertoli cells (42 %), and Leydig cells (43 %) in the animals exposed to the high dose of Na-MBS in comparison to the control groups (Figure 2(Fig. 2)). 

However, the cell population significantly recovered in the rats treated with Na-MBS (intermediate and high doses) plus CUR compared to those that received Na-MBS without CUR therapy.

### Qualitative evaluation

**Figure 3:**
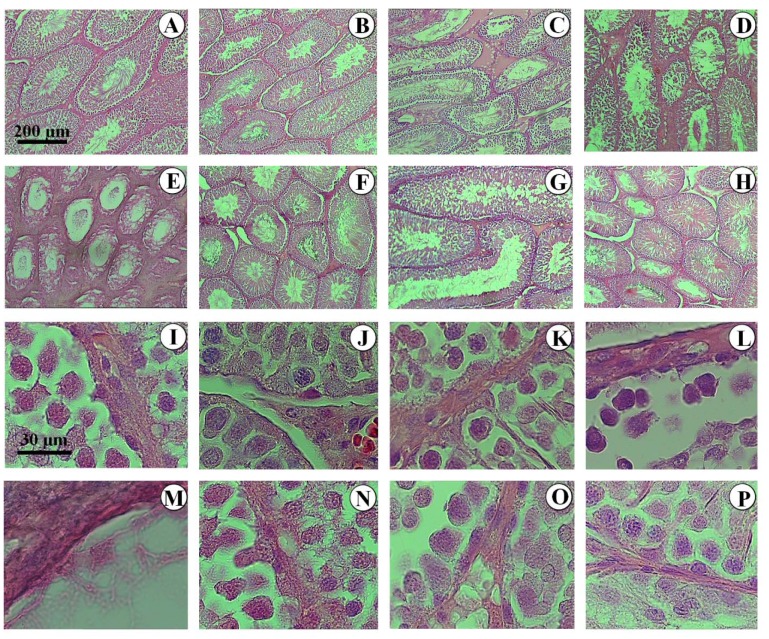
Photomicrograph of the testicles' histology in different groups. In the control (A, I), curcumin (B, J), and low dose of Na-MBS (C, K) groups, the tubules and testicular cells maintained normal form. In the animals treated with intermediate (D, L) and high doses of Na-MBS (E, M), the tubules seemed atrophic, the interstitial tissue increased, epithelium height decreased, and many testicular cells were lost. No changes were observed in the low dose of Na-MBS+CUR group (F, N). In the rats treated with intermediate (G, O) and high doses of Na-MBS+CUR (H, P), the protective effects of curcumin considerably restored the tubules, interstitium, and epithelial cells.

Comparison of different groups regarding testicle histology is depicted in Figure 3(Fig. 3). Accordingly, tubules and testicular cells maintained their normal form in the control groups and those treated with CUR and low dose of Na-MBS+CUR. However, the tubules seemed atrophic in the animals treated with intermediate and high doses of Na-MBS. In addition, the volume of the interstitial tissue increased, while the height of the epithelium decreased in these groups. The decrease occurred in the volume of germinal epithelium as well as Leydig cells was yet another finding. On the other hand, an improvement was observed in the groups treated with intermediate and high doses of Na-MBS plus CUR. Based on the micrographs of Figure 3(Fig. 3), not only CUR restored the massive changes in the tubules and interstitium, but also it reverted the decline in the number of spermatogenic, Sertoli, and Leydig cells.

## Discussion

The first step of the present study manifested the effects of the ingestion of different doses of Na-MBS on the testicle. The second step revealed the protective effects of CUR in this context. Based on the results, ADI of Na-MBS did not affect the testicle. However, the amount of Na-MBS that can enter human body is directly related to dietary habits. In other words, more consumption of processed or preserved foods is accompanied with higher possibility of adverse effects of Na-MBS. It is obvious that the amount of daily intake of Na-MBS could not be precisely determined in different societies. 

Spermatozoa analysis allows researchers to evaluate the performance of the testicle. Reduction of spermatozoa count, motility, and normal morphology in the current study was in accordance with other researches. Previous studies also reported some toxic impacts of sulfites on the reproductive system (Cabré et al., 1990[7]; Woo et al., 2003[23]). “Sulfating agents” are harmful because they release SO_2_, a toxin to mammal reproductive system, in the tissue. As mentioned by Meng and Bai (2004[14]), SO_2_ exposure could cause oxidative damage to testicles of male mice. Recently, Shekarforoush et al. (2015[19]) also reported that normal morphology, count, and motility of spermatozoa decreased in rats treated with 100 and 260 mg/kg body weight of Na-MBS for 28 days (Shekarforoush et al., 2015[19]). The findings of the present study revealed that treating the rats even with lower doses (7 and 70 mg/kg/ day) for a longer period (7 weeks) led to toxic effects, as well. The reduction in spermatozoa parameters is a consequence of the reduction in germinal epithelial cells after exposure to Na-MBS. Sertoli supporting cells are the “nurse” cells of the epithelium and help in the process of spermatogenesis. Loss of the nurse cells in Na-MBS-treated animals could be considered to be one of the reasons for the loss of spermatogenic cells due to deficiency in its supportive functions. Another possible mechanism might be the direct effects of oxidative damage. Oxidative stress induced by Na-MBS on the testicle has been described by a previous study (Adebayo and Adenuga, 2012[2]). Measurement of the levels of some oxidant and antioxidant enzymes in their survey showed a significant increase in malondialdehyde and superoxide dismutase in Na-MBS-treated rats. On the other hand, a significant decline was observed in the activity of catalase in that research. However, the level of glutathione was not considerably affected in their study (Adebayo and Adenuga, 2012[2]). Generally, limited amount of sulfite can be detoxified by human body using sulfite oxidase which is an enzyme (located in the mitochondria) that oxidizes sulfite to sulfate (Cabré et al., 1990[7]; Woo et al., 2003[23]). In the case of exposure to excessive amount of sulfite in tissues with low levels of sulfite oxidase (including testicle), cellular toxicity can be evoked. 

Furthermore, the current study results documented the protective effects of CUR on spermatozoa parameters and testicular cells in the animals treated with Na-MBS. Previous studies also reported the protective effects of CUR under different conditions. For instance, a previous study reported that CUR could protect spermatozoa count, motility, and morphology from adverse effects of Lindane (an organochlorine chemical agent) in male rats (Sharma and Singh, 2010[18]). Another earlier study also claimed that CUR supplementation could ameliorate testicular injury induced by phthalate (a plasticizer agent) in rats (Abd El-Fattah et al., 2016[1]). In addition, according to previous studies, CUR had an anti-apoptotic effect and could improve spermatogenesis in testicles of mice exposed to scrotal heat stress (Aktas et al., 2012[3]; Lin et al., 2015[13]). Therefore, the protective effects of CUR against Na-MBS in the present study might be explained by its antioxidant and anti-apoptotic effects reported in the previous studies (Takhtfooladi et al., 2015[20]; Sharma and Singh, 2010[18]).

The acceptable daily intake of Na-MBS for 7 weeks did not affect on testicular parameters. CUR (100 mg/kg/day) could prevent structural impairments of testicles in the rats induced by Na-MBS (7 and 70 mg/kg/day). 

## Acknowledgements

This work was financially supported by grant No. 23-2-555 from Yasuj University of Medical Sciences, Yasuj, Iran. This article was a part of the thesis written by Zahra Honarmand, MSc student of Anatomy. Hereby, the authors would like to thank Ms. A. Keivanshekouh at the Research Improvement Center of Shiraz University of Medical Sciences for improving the use of English in the manuscript.

## Conflict of interest

The authors declare no conflict of interest.

## Figures and Tables

**Table 1:**
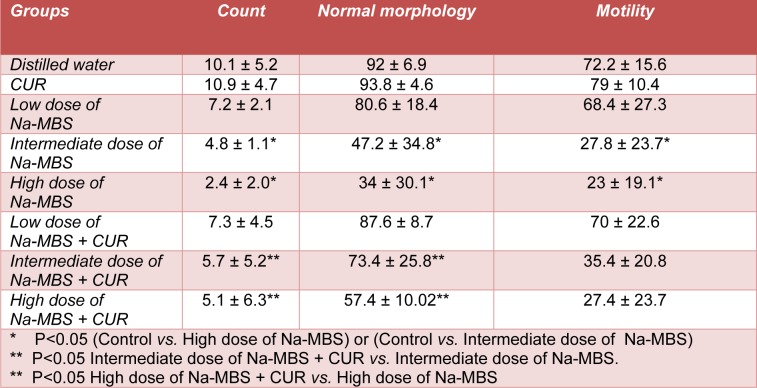
Mean ± standard deviation of the spermatozoa count (×10^6^), normal morphology (%) motility (%) for the groups of distilled water, CUR, Na-MBS (low, intermediate and high doses), Na-MBS (low, intermediate and high doses) + CUR
